# Creation of Pd/Al_2_O_3_ Catalyst by a Spray Process for Fixed Bed Reactors and Its Effective Removal of Aqueous Bromate

**DOI:** 10.1038/srep41797

**Published:** 2017-02-02

**Authors:** Yu Gao, Wuzhu Sun, Weiyi Yang, Qi Li

**Affiliations:** 1 Environment Functional Materials Division, Shenyang National Laboratory for Materials Science, Institute of Metal Research, Chinese Academy of Sciences, Shenyang 110016, P. R. China; 2 Institute of Materials, China Academy of Engineering Physics, Mianyang 621900, P. R. China; 3 School of Materials Science and Engineering, Shandong University of Technology, Zibo 255000, P. R. China

## Abstract

Palladium nanoparticles were grown on sub-millimeter activated Al_2_O_3_ particle support by spraying H_2_PdCl_4_ solution evenly onto the support, followed with a thermal reduction under H_2_ atmosphere. Compared with its counterpart created by the conventional impregnation method, the Pd/Al_2_O_3_ catalyst created by the spray process could enrich the existence of active Pd nanoparticles on the surface of the catalyst support and increase their degree of dispersion, resulting in a much higher activity in the catalytic reduction of bromate in water. The effect of Al_2_O_3_ support particle size on the bromate removal rate was also investigated, which demonstrated that smaller support particle size could have higher activity in the catalytic reduction of bromate in water because of its larger exposed surface. This Pd/Al_2_O_3_ catalyst could be easily used in the fixed bed reactor due to its large support size and demonstrated excellent stability in the catalytic reduction of bromate in mineral water. This Pd/Al_2_O_3_ catalyst also exhibited a good catalytic reduction performance on azo dyes as demonstrated by its effective catalytic hydrogenation of methyl orange. Thus, catalysts prepared by the spray method developed in this work could have the potential to be used in fixed bed reactors for various water treatment practices.

Although bromate is not normally present in natural water, it could be readily formed as a toxic disinfection by-product during the drinking water disinfection with ozone when bromide ion is present in the raw water^1^,^2^. As a Group 2B substance defined by the International Agency for Research on Cancer (IARC)^3^,^4^, bromate had been recognized as having sufficient evidence of carcinogenicity in lab animals and potential carcinogenicity in human. Thus, World Health Organization (WHO) had suggested a regulatory maximum contaminant level (MCL) of 10 ppb for bromate in drinking water, and it had been adopted by many countries worldwide, including USA, European Union, Japan, and China^5^,^6^. An even more rigid MCL of 3 ppb had been established by European Union for ozonated natural mineral water and spring water^7^.

To meet the drinking water standard, various processes had been developed to treat bromate in drinking water, including the removal of bromate precursor, minimization of its formation during the ozone disinfection, and removal of it after its formation^8^,^9^. Among them, the removal of bromate after its formation is believed to be a more feasible approach^9^,^10^, and a variety of techniques have been suggested, including ultraviolet irradiation^11^,^12^, arc discharge^13^, adsorption^14^,^15^, membrane processes^16^, ion exchange^8^,^17^, coagulation^16^, chemical reduction^18^,^19^, electrochemical reduction^20^,^21^, photocatalytic reduction^22^,^23^, and biological methods^24^. However, most of these techniques encountered limitations such as low efficiency, secondary contamination, and concentrated waste streams. Thus, it is highly desirable to develop more effective approaches for the removal of bromate from drinking water.

As a fast, efficient and clean process, the catalytic hydrogenation has been considered as a promising approach to treat harmful reducible contaminants in water since the first report on the catalytic hydrogenation of nitrate in water by Vorlop and Tacke in 1989^25^. Because it could be conducted under mild conditions without the production of contaminated disposals, this process has been extensively investigated for the removal of various contaminants from water, including nitrate^26^,^27^, nitrite^28^, perchlorate^29^,^30^, chlorinated organic contaminants^31^, chromium (VI)^32^, and azo dyes^33^. In 2010, Chen *et al*.^34^ reported the first catalytic reduction of bromate with noble metal catalysts loaded on various powder supports. Since then, efforts had been made to develop various catalysts for the catalytic reduction of aqueous bromate with encouraging results^35^,^36^,^37^,^38^,^39^. However, these catalysts could not be efficiently separated from treated water due to their powder form supports, and their dispersion into the aqueous environment may result in secondary pollution and a high cost due to their active components of expensive noble metals. To solve this problem, Pd catalysts on magnetically separable powder supports were developed^40^,^41^, which could provide a more efficient removal for these catalysts after their catalytic reduction of aqueous bromate. But they still could not be adopted in the most commonly used fixed-bed reactors in the water treatment industry^42^.

Recently, Pd was loaded onto carbon nanofibers grown on various macroscopic supports (such as sintered metal fibers or monoliths)^43^,^44^,^45^,^46^, which could be utilized in the fixed-bed reactors for the catalytic bromate reduction. However, their synthesis processes were generally complex and involved the use of expensive instruments, which largely increased their cost. Furthermore, their loading of palladium relied on the conventional impregnation method, which could load active palladium component into mesopores in the deep inside of these porous supports. Thus, the contact efficiency of reactants with active components decreased and the mass transfer limitation increased in the reaction, adverse to the improvement of the catalytic bromate reduction efficiency. Therefore, new methods should be developed to simplify the synthesis process, lower the production cost, and enrich the presence of active components on the catalyst surface for creating high efficient catalysts to be utilized in fixed-bed reactors for their potential water treatment practice.

In this work, a Pd/Al_2_O_3_ catalyst was developed by spraying H_2_PdCl_4_ solution evenly onto the sub-millimeter activated Al_2_O_3_ particle support, followed with a thermal reduction at 300 °C under H_2_ atmosphere. Compared with its counterpart created by the conventional impregnation method, it demonstrated much higher activity in the catalytic reduction of bromate in water because more active Pd components were enriched on its surface and their degree of dispersion increased, effectively enhancing the contact efficiency of reactants with Pd and minimizing the mass transfer limitation in the reaction. Both batch and continuous flow experiments were conducted for the catalytic bromate reduction. The study of the Al_2_O_3_ support particle size effect on the bromate removal demonstrated that smaller support particle size could have higher activity in the catalytic bromate reduction. The Pd/Al_2_O_3_ catalyst demonstrated a superior bromate removal capability during a continuous flow catalytic bromate reduction experiment in a lab-prepared, packed fixed-bed tube reactor, in which it completely removed bromate from natural mineral water of over 24000 bed volumes (BVs) during a continuous operation for 10 days. The Pd/Al_2_O_3_ catalyst also exhibited a good catalytic reduction performance on azo dyes as demonstrated by its effective catalytic hydrogenation of methyl orange.

## Results and Discussion

### Structure and morphology of Pd/Al_2_O_3_ catalysts

**Figure 1:**
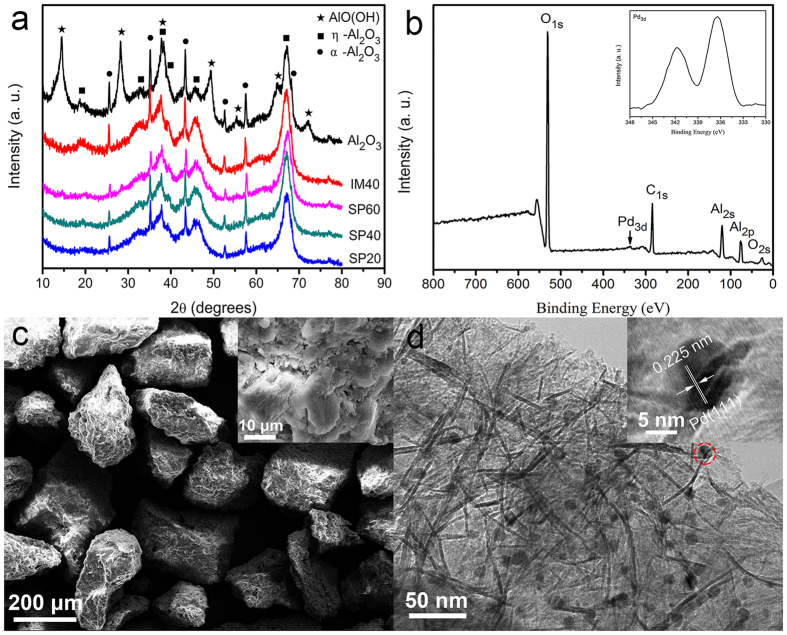
(**a**) XRD patterns of activated Al_2_O_3_ and Pd/Al_2_O_3_ catalysts with a series of grounded activated Al_2_O_3_ particle sizes. (**b**) to (**d**) The representative XPS survey spectrum (**b**), SEM image (**c**), and TEM image of SP60 sample. (Note: The insert image in Fig. 1c shows the SEM image with a higher magnification, and the insert image in Fig. 1d shows the corresponding HRTEM image of the Pd nanoparticle with red circle.).

Figure 1a shows XRD patterns of activated Al_2_O_3_ and Pd/Al_2_O_3_ catalysts with a series of grounded activated Al_2_O_3_ particle sizes. For activated Al_2_O_3_, diffraction peaks belonging to *α*-Al_2_O_3_ (JCPDS No. 81-1667), *η*-Al_2_O_3_ (JCPDS No. 77-0396), and AlO(OH) (JCPDS No. 74-1895) could be clearly identified. For Pd/Al_2_O_3_ catalysts, the diffraction peaks of AlO(OH) could not be observed because activated Al_2_O_3_ was dehydrated in the calcination step during the Pd/Al_2_O_3_ catalyst synthesis process. No diffraction peaks of Pd could be identified in XRD patterns of these Pd/Al_2_O_3_ catalysts, which could be attributed to the low Pd-loading amount in them. ICP measurements demonstrated that the Pd-loading amount in Pd/Al_2_O_3_ catalysts prepared by the spray method was ~0.79 wt%, 0.81 wt%, and 0.88 wt% for SP20, SP40, and SP 60 samples, respectively, and the Pd-loading amount in IM40 sample prepared by the conventional impregnation method was ~0.94 wt%, which were too low to be detected by XRD^34^.

To examine the existence status of palladium on these Pd/Al_2_O_3_ samples, XPS analysis was conducted. Figure 1b shows the representative XPS survey spectrum of SP60 sample, which clearly demonstrated the existence of Al, O, and Pd. Due to the widespread presence of carbon in the environment, C 1 *s* peak could also be observed clearly in this XPS survey spectrum. The insert image in Fig. 1b shows the high-resolution XPS scan over Pd 3*d* peaks of SP60 sample. The binding energy of Pd 3*d*_3/2_ and 3*d*_5/2_ peaks was determined at ~341.3 eV and ~336 eV, respectively. Similar results were observed on other Pd/Al_2_O_3_ samples, which demonstrated clearly that metallic Pd^32^,^40^ was successfully loaded as the active component on sub-millimeter activated Al_2_O_3_ particle support by the spray method we developed after the thermal reduction at 300 °C under H_2_ atmosphere.

Figure 1c shows the SEM image of SP60 sample, which demonstrated the irregular shape, sub-millimeter size, rough surface, and porous structure of the catalyst support. The insert image in Fig. 1c shows its SEM image with a higher magnification, which clearly demonstrated that inter-connected macropores existed throughout the support, beneficial for liquid transport. Figure 1d shows the TEM image of SP60 sample, which demonstrated that dark Pd nanoparticles with the average particles size of several nm were distributed relatively evenly on the Al_2_O_3_ support. The insert image in Fig. 1d shows the corresponding high resolution TEM (HRTEM) image of the Pd nanoparticle with the red circle. One set of lattice planes could be clearly observed with the *d*-spacing at ~0.225 nm, corresponding to the (111) plane of metallic Pd (JCPDS No. 88-2335). This observation was consistent with the XPS analysis result. TEM and HRTEM images of SP20, SP40 and IM40 samples were demonstrated in Fig. S1 in the supplementary information, which showed similar results as that of SP60 sample.

### Surface Properties of Pd/Al_2_O_3_ catalysts

**Figure 2:**
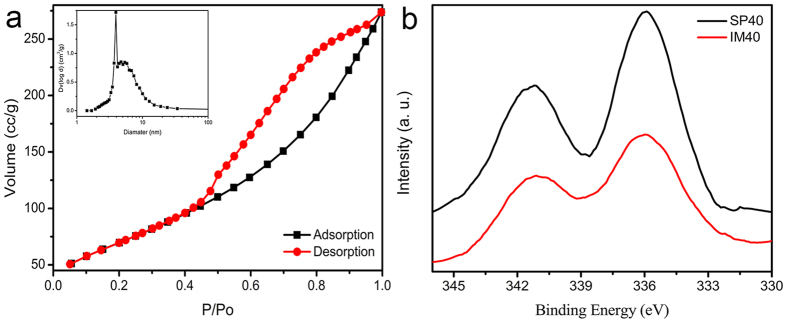
(**a**) The representative N_2_ adsorption/desorption isotherms of SP60 sample. (Note: Insert image in Fig. 2a shows the pore size distribution of SP60 sample calculated from the desorption data with BJH model.) (**b**) High resolution XPS scans over Pd 3*d* peaks of SP40 and IM40 samples, respectively.

To examine the specific surface area and pore structure of these Pd/Al_2_O_3_ samples, N_2_ adsorption/desorption isotherm measurements were conducted, which demonstrated that they were very similar. Figure 2a shows the representative N_2_ adsorption/desorption isotherms of SP60 sample. The shape of the BET curve is the characteristics of mesoporous structure^47^. The BET surface specific area of SP60 sample was found to be ~259.1 m^2^ · g^−1^. The insert image in Fig. 2a shows the pore size distribution of SP60 sample calculated from the desorption data with BJH model, which suggested that most pores in it were mesopores and the average pore diameter was determined to be ~6.497 nm. The specific pore volume of SP60 sample was measured to be ~0.4208 cm^3^ · g^−1^. The BET specific surface area, average pore diameter, and specific pore volume of these Pd/Al_2_O_3_ samples were summarized in Table 1, compared with that of their activated Al_2_O_3_ supports. The loading of Pd nanoparticles onto sub-millimeter activated Al_2_O_3_ particles caused a moderate decrease of their specific surface areas, while it had not much effect on the pore structures of their supports, especially for samples prepared by the spray method developed in this work because the Pd-loading was more concentrated on the exposed macroscopic surface of supports by the spray method.

To examine the effect of different Pd-loading methods on the Pd existence on the surface these Pd/Al_2_O_3_ catalysts, high resolution XPS scans over Pd 3*d* peaks were conducted on SP40 and IM40 samples as shown in Fig. 2b. For both samples, the binding energy of Pd 3*d*_3/2_ and 3*d*_5/2_ peaks was determined at ~341.3 eV and ~336 eV, respectively, which demonstrated clearly metallic Pd^32^,^40^ was successfully loaded by both methods after the thermal reduction at 300 °C under H_2_ atmosphere. However, the Pd XPS signal intensities of these two samples were quite different. It was clear that SP40 sample had a much stronger Pd XPS signal intensity than IM40 sample, although ICP measurement results demonstrated that more Pd (~0.94 wt%) was loaded on IM40 sample by the conventional impregnation method than that on SP40 sample (~0.81 wt%) by the spray method developed in this work. The surface Pd/Al atomic ratio of SP40 sample determined by XPS was ~1:240, which was much higher than that of IM40 sample of ~1:474. Thus, Pd could be enriched on the surface of these sub-millimeter activated Al_2_O_3_ particle supports by the spray methods we developed than the conventional impregnation method, which should be beneficial for their catalytic reduction efficiency by reducing the mass transport limitation and subsequently facilitating the catalytic reduction process.

The CO chemisorption analysis results showed that the degree of dispersions of Pd nanoparticles in SP20, SP40, SP60 and IM40 samples was ~13.3%, 20.9%, 21.7% and 14.0%, respectively, and the corresponding average particle sizes of Pd nanoparticles in these Pd/Al_2_O_3_ catalysts was ~8.44, 5.36, 5.16 and 8.01 nm, respectively. The comparison between SP40 and IM40 samples clearly demonstrated that the spray method could load Pd nanoparticles with larger degree of dispersion and smaller particle size than the impregnation method, which should be beneficial for the catalytic reduction efficiency due to the enhancement of the contact efficiency of reactants with Pd. In addition, the degree of dispersion of Pd nanoparticles on Pd/Al_2_O_3_ catalysts prepared with the spraying method increased with the particle size decrease of Al_2_O_3_ catalyst support.

### Catalytic bromate reduction in the batch reactor

**Figure 3:**
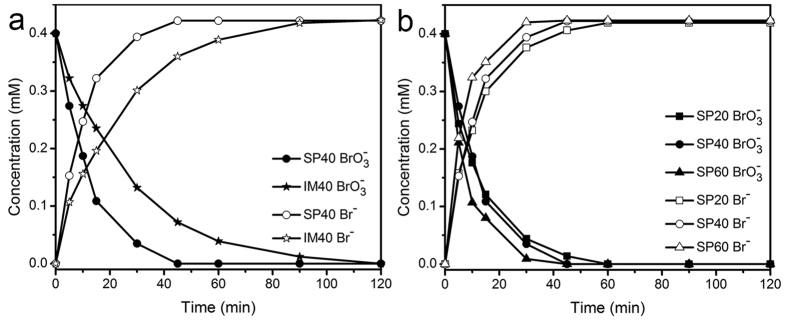
(**a**) The catalytic bromate reduction by SP40 and IM40 samples in the batch reactor, respectively. (**b**) The catalytic bromate reduction by SP20, SP40, and SP60 samples in the batch reactor, respectively.

Figure 3a compares the catalytic bromate reduction by SP40 and IM40 samples in the batch reactor. The sum of bromate and bromide concentrations during the reaction process was ~0.4 mM for both catalysts, which was equal to the initial bromate concentration in the solution. This observation indicated that bromide was the only catalytic reduction product by both catalysts in the present study^34^. After just 45 min reaction, the bromate concentration dropped from ~0.4 mM (51 ppm) to zero (below the detection limit of ~1 ppb) by the treatment of SP40 sample, representing a 100% conversion ratio, and its initial bromate reduction rate was determined at 36.7 mmol_Bromate_ · h^−1^ · g_cat_^−1^. For IM40 sample, however, the complete bromate reduction required 120 min treatment, and its initial bromate reduction rate was determined at 25.7 mmol_Bromate_ · h^−1^ · g_cat_^−1^. This comparison clearly demonstrated the importance of the Pd-loading method for the catalytic reduction performance of these Pd/Al_2_O_3_ catalysts. SP40 and IM40 samples were created by loading Pd nanoparticles onto the same Al_2_O_3_ catalysts support with the spray method and the conventional impregnation method, respectively, and the Pd-loading amount in IM40 sample (0.94 wt%) was even higher than that of SP40 sample (0.81 wt%). However, the catalytic bromate reduction performance of IM40 sample was obvious worse than that of SP40 sample under the same experimental conditions, which could be attributed to the surface enrichment of active Pd component and the degree of dispersion increase of Pd nanoparticles in SP40 sample. When the conventional impregnation method was used to load Pd on porous Al_2_O_3_ support, the Pd precursor solution could enter and then fill up the mesopores in the deep inside of the porous support. Thus, many Pd particles were created in these mesopores during the following drying, calcination, and reduction process, which could not be utilized efficiently during the catalytic bromate reduction. The spray method, by contrast, could place more Pd precursor solution more evenly on the surface or macropores of the Al_2_O_3_ support, which prevented the diffusion of Pd precursor solution into the mesopores in the deep inside. Thus, it could enrich the Pd existence on the accessible catalyst surface and increase its degree of dispersion, which resulted in the observed catalytic reduction performance enhancement.

Figure 3b compares the catalytic bromate reduction by SP20, SP40, and SP60 samples in the batch reactor. All three samples were prepared by the spray method, while their catalyst support had different sizes of 20~40, 40~60, and 60~100 meshes, respectively. All three samples could completely reduce bromate to bromide efficiently, while SP60 sample had the best performance. The specific surface area of these catalysts was mainly determined by the mesoporous structure of their Al_2_O_3_ supports, which did not change much during the grinding process to obtain supports with different particle sizes. Although these three samples had similar specific surface area values, smaller one could have a larger exposed surface to contact and immobilize Pd precursor during the spray and subsequent drying process, leading to more Pd enrichment on the support surface (the surface Pd/Al atomic ratio of SP60 sample at ~1:198) and its larger degree of dispersion (SP60 sample at ~21.7%) after the thermal reduction process. Thus, SP60 sample demonstrated the best performance among them. Table 2 compares the catalytic bromate reduction activity between SP60 sample and various Pd-based catalysts reported in literature^34^,^35^,^36^,^40^,^41^,^42^,^43^,^44^,^45^,^46^, and their initial bromate reduction rate was calculated based on the amount of active Pd component. It demonstrated clearly that our SP60 sample had a superior bromate reduction performance among these Pd-based catalysts.

As applied in previous studies of the reduction of bromate^34^,^40^, the pseudo-first-order kinetic model was used to fit the catalytic bromate reduction data in this work. The pseudo-first-order kinetic model could be defined by Eq. (1) and its integrated form was given in Eq. (2):


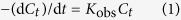



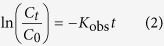


where *C*_*t*_ is the bromate concentration at treatment time *t, C*_0_ is the initial bromate concentration, and *K*_obs_ is the observed pseudo-first-order rate constant. Table 3 summaries the kinetics parameters obtained in fitting the experimental data. The applicability of the pseudo-first-order rate model was quantified by the square of the correlation coefficient *R (R*^2^). *R*^2^ for SP20, SP40, SP60, and IM40 samples were determined at 0.999, 0.994, 0.980, and 0.999, respectively. The closeness of *R*^2^ to 1 indicated that the pseudo-first-order kinetic model fitted the experimental data of the catalytic bromate reduction by these Pd/Al_2_O_3_ catalysts accurately. The *K*_obs_ for SP20, SP40, SP60, and IM40 samples were determined at 0.0717 min^−1^, 0.0822 min^−1^, 0.1255 min^−1^, 0.0394 min^−1^ respectively, and SP60 sample had the largest *K*_obs_ among these Pd/Al_2_O_3_ catalysts.

### Catalytic bromate reduction in the fixed-bed reactor with lab-prepared water sample

**Figure 4:**
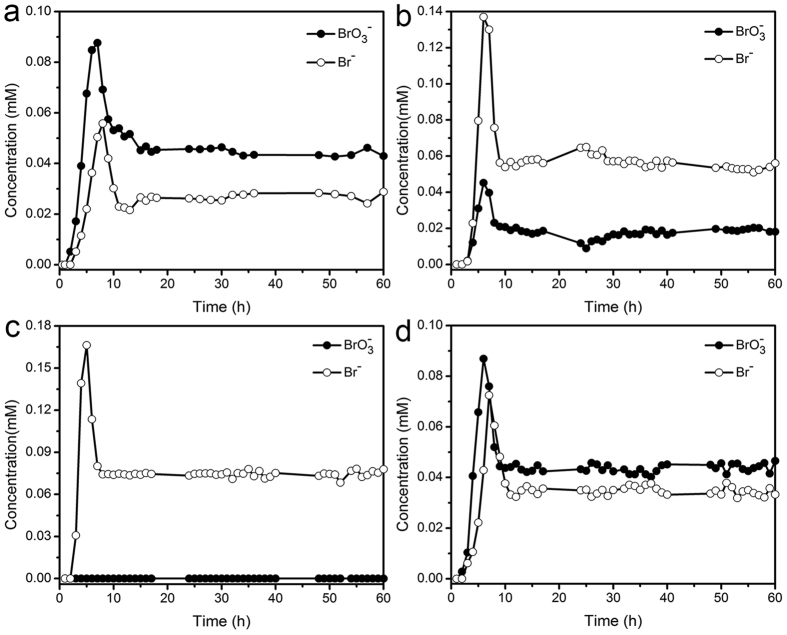
Bromate and bromide concentrations in the effluent at different treatment times for fixed-beds of SP20 (**a**), SP40 (**b**), SP60 (**c**), and IM40 (**d**) samples, respectively.

To examine their potential for industrial applications, the continuous flow catalytic bromate reduction experiments were conducted in a lab-prepared, packed fixed-bed tube reactor. In order to avoid issues related to the back mixing of the solution, the diameter and height of the fixed-bed were designed to be more than 10 times and 100 times to the diameter of the catalyst support particle, respectively^42^. Figure 4 shows the experiment data of bromate and bromide concentrations in the effluent at different treatment times for fixed-beds of SP20, SP40, SP60, and IM40 samples, respectively. The test solution was prepared by RO water with 0.078 mM bromate, and Table 4 summarizes its water quality data. Besides the additionally added bromate, Cl^−^, NO_3_^−^, and SO_4_^2−^ ions were also found in it with the similar level of concentrations as bromate. The loading rate was ~2.83 L · m^−2^ · s^−1^ (10.19 m · h^−1^), and the empty bed contact times (EBCT) was only ~35 sec. It could be found that after an initial period within the first 10 h, the bromate and bromide concentrations in the effluent reached their equilibrium state and kept mostly constant till the end of the experiments.

In the initial period, adsorption, ion exchange and catalytic reduction all happened, which resulted in an interesting bromate and bromide concentration change observed in the effluent with the increase of experiment time. In the first ~2 h, bromate and bromide produced by the catalytic reduction were completely adsorbed onto the surface of these catalyst fixed-beds, so they could not be detected in the effluent. Next, from ~2 h to ~6 h, the adsorption of bromate and bromide approached to the saturation status, and more and more bromate and bromide remained in water and their concentrations gradually increased. Co-existed ions in the solution, such as sulfate, nitrate and chloride could exchange with adsorbed bromate and bromide due to their stronger affinity to these catalyst fixed-beds, and the concentrations of bromate and bromide in the effluent could continuously increase so that their sum could be even higher than the bromate concentration in the test solution. Then, from ~6 h to ~10 h, the concentrations of bromate and bromide in the effluent decreased because the ion exchange decreased as fewer and fewer bromate and bromide were still adsorbed on the surface of these catalyst fixed-beds. Finally, as the adsorption and desorption by the ion-exchange of bromate and bromide reached equilibrium, the change of their concentrations in the effluent stopped to reach the equilibrium state till the end of the experiments.

For all these Pd/Al_2_O_3_ catalysts, the sum of the equilibrium concentrations of bromate and bromide in the effluent was ~0.078 mM, equal to the bromate concentration of the test solution. This observation suggested that bromate was successfully reduced to non-toxic bromide during the flow of test solution through the fixed-bed reactor by its contact with these Pd/Al_2_O_3_ catalysts and H_2_, and these Pd/Al_2_O_3_ catalysts had a good stability in the packed bed mode and could be effective for a long time. The equilibrium bromate concentration in the effluent through the fixed-bed of SP40 was ~0.017 mM, while that of IM40 was ~0.044 mM. Thus, bromate reduced by the fixed-bed of SP40 was almost twice as that by the fixed-bed of IM40. The comparison between SP40 and IM40 samples clearly demonstrated that SP40 also had a much better performance than IM40 in the packed bed mode, which could be attributed to the effect of their different Pd-loading methods. With such a fast EBCT of ~35 sec, it was difficult for bromate in the test solution to diffuse into mesopores of IM40 sample in its deep inside of the porous Al_2_O_3_ support to react with Pd there, while it was much easier for bromate in the test solution to react with enriched Pd on the surface of SP40 sample. For the series of samples prepared by the spray method, the equilibrium bromate concentration in the effluent through the fixed-bed of SP20, SP40, and SP60 was ~0.045 mM, ~0.017 mM, and zero, respectively. Thus, catalysts with smaller support particle size had the better catalytic bromate reduction performance, which was the same as their batch experiment results. Besides the reason that smaller support particles could have larger exposed surface for Pd-loading, smaller support particles could have better intraparticle mass transfer, which also contributed to their better catalytic reduction performance. Among them, SP60 sample demonstrated a superior bromate reduction capability. No bromate was detected in the effluent through the whole period of the continuous flow catalytic bromate reduction experiment by the fixed-bed of SP60.

### Catalytic bromate reduction in the fixed-bed reactor of SP60 with natural mineral water sample

**Figure 5:**
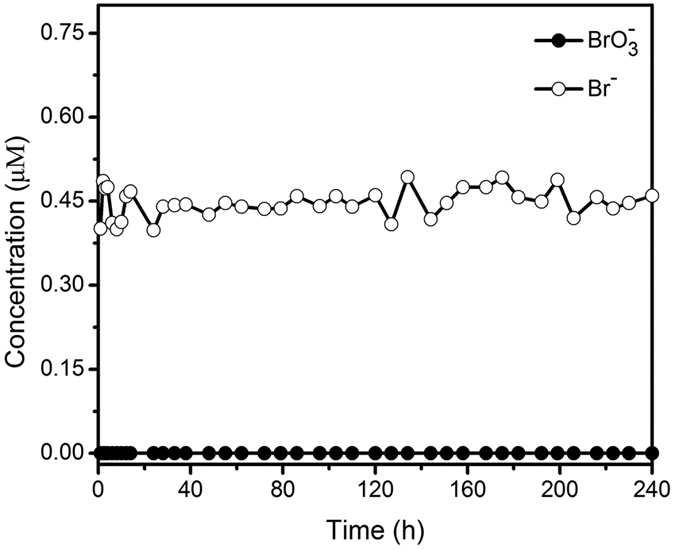
Bromate and bromide concentrations in the effluent of natural mineral water sample at different treatment times up to 10 days for the fixed-bed of SP60 sample.

In the industry, natural mineral water generally goes through the ozonation disinfection process before it is bottled or barrelled during its production. It usually contains bromate exceeding the drinking water limitation if bromide is present in the raw water. Both the complexity of substances in natural mineral water and the effect of residual ozone must be considered when dealing with the application of a catalyst to treat bromate contamination in natural mineral water production. Thus, the bromate removal performance of SP60 sample in the fixed-bed reactor was tested with natural mineral water sample for a continuous operation of 10 days. Bromate was added into the natural mineral water sample with the concentration of 0.40 μM (51 ppb), which was in the high end of bromate concentration found in drinking water after the ozonation disinfection process^9^. The water quality data of the natural mineral water sample could be found in Table 4, which clearly demonstrated the existence of various cations and anions with much higher concentrations (~22 to 8700 times) than that of the additionally added bromate. For the introduction of residual ozone into the natural mineral water sample, it was bubbled with an ozone flow of 3.5 L · min^−1^ for 2 h in every 50 kg water before being used. The loading rate was ~2.83 L · m^−2^ · s^−1^ (10.19 m · h^−1^), and the empty bed contact times (EBCT) was only ~35 sec. Figure 5 shows the bromate and bromide concentrations in the effluent at different treatment times up to 10 days for the fixed-bed of SP60 sample, which clearly demonstrated that bromate was completely removed during the whole treatment time from the natural mineral water sample, although it contained various competition ions at high concentration and residual ozone. Thus, our SP60 sample demonstrated a good potential for the catalytic reduction of bromate in the drinking water production industry.

### Catalytic methyl orange reduction by SP60 sample

**Figure 6:**
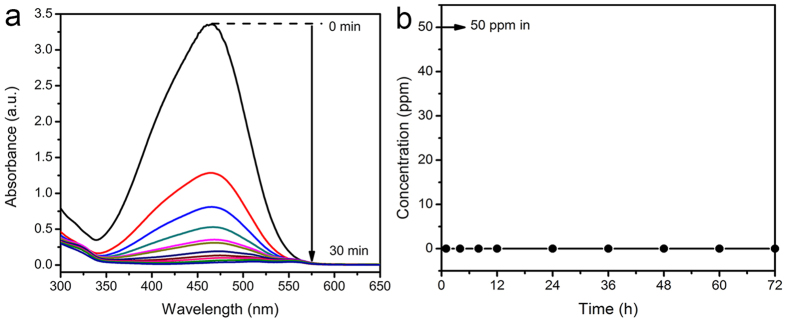
(**a**) The representative light absorption spectra of MO solutions at different treatment times by SP60 sample. (**b**) MO concentration in the effluent at different treatment times up to 3 days for the fixed-bed of SP60 sample.

These Pd/Al_2_O_3_ catalysts could also be utilized to treat other harmful reducible contaminants in water. For example, water pollution by dyes from the textile industries has become a serious problem in many countries^48^,^49^. The removal of dyes from wastewater is a challenge to relevant industries because commonly used synthetic dyes, for example azo dyes, are stable compounds and difficult to be treated in the water treatment practice^48^. The reduction of azo dyes could reduce the chromaticity color of wastewater, which subsequently could increase the self-cleaning capability of water and reduce the difficulty of the azo dye degradation. In this study, methyl orange (MO) was used as the model organic pollutant to examine the catalytic reduction capability of SP60 sample on azo dyes. Figure 6a shows the representative light absorption spectra of MO solutions at different treatment times by SP60 sample (0.05 g · L^−1^) under an H_2_ flow (200 mL · min^−1^), and the initial MO concentration was 50 ppm. With the increase of the treatment time, the light absorption of MO solution (at ~465 nm) decreased steadily, indicating the continuous decrease of MO concentration in the solution. After just 30 min, the light absorption of MO solution dropped to near zero, indicating a near complete reduction of MO. Thus, SP60 sample demonstrated a good catalytic reduction capability on MO. The MO catalytic reduction performance of SP60 sample in the fixed-bed reactor was also tested for a continuous operation of 3 days. The loading rate of MO solution was ~2.83 L · m^−2^·s^−1^ (10.19 m · h^−1^), and the empty bed contact time (EBCT) was also only ~35 sec. No color was observed in the effluent during the whole experiment time even with such a short EBCT. Figure 6b shows the MO concentration in the effluent at different treatment times up to 3 days for the fixed-bed of SP60 sample, which clearly demonstrated that MO was completely reduced during the whole treatment time.

In summary, a spray method was developed for Pd-loading onto sub-millimeter activated Al_2_O_3_ porous particle support, which resulted in the surface enrichment of active Pd nanoparticles and their larger degree of dispersion compared with the conventional impregnation method. By this means, the contact efficiency of reactants with catalysts could be enhanced, and the mass transfer limitation in the reaction could be minimized. Thus, Pd/Al_2_O_3_ catalyst prepared by the spray method demonstrated much higher activity in the catalytic reduction of bromate in water than its counterpart created by the conventional impregnation method with even higher Pd-loading amount. These Pd/Al_2_O_3_ catalysts could be easily adopted in the fixed bed reactor due to its large support size, which was beneficial for their potential usage in the water treatment practice. By modulating the size of Al_2_O_3_ particle support, Pd/Al_2_O_3_ catalyst with a superior bromate reduction capability was developed, which completely removed bromate from natural mineral water sample in the fixed-bed reactor for a continuous run of 10 days. It could also be used for the catalytic reduction of other harmful reducible contaminants in water as demonstrated by its effective catalytic reduction of methyl orange. Thus, the spray method developed in this work may be readily adopted to prepare catalysts with enriched existence of active components on their surface, and these catalysts developed could have the potential to be used in fixed bed reactors for various water treatment practices.

## Methods

### Chemicals and materials

All chemicals were of reagent grade and used without further purification. Palladium dichloride, hydrochloric acid, sodium bromate, sodium bromide, and activated Al_2_O_3_ in the form of porous pellets were obtained from Sinopharm Chemical Reagent Co., Ltd (Shanghai, P. R. China). Hydrogen gas (99.99%) was produced with a hydrogen generator (Shenyang Guangzheng Co., Shenyang, P. R. China).

### Synthesis of Pd/Al_2_O_3_ catalysts

Activated Al_2_O_3_ pellets were first ground and screened to obtain particles with desirable diameters (20~40 mesh, 40~60 mesh, and 60~100 mesh), followed by washing with deionized (DI) water and oven drying at 120 °C for 12 h. Next, palladium were loaded on these sub-millimeter activated Al_2_O_3_ particle supports by spraying H_2_PdCl_4_ solution (10.5 mL containing 0.167 g PdCl_2_) evenly onto the support (10 g) through a spray bottle for three times. After each spraying, the sample was dried immediately at 100 °C for 1 h before the next spraying. Then, the sample was dried at 100 °C for 12 h, calcinated at 450 °C for 2 h in air, and subsequently reduced at 300 °C under H_2_ atmosphere for 30 min to obtain the desired Pd/Al_2_O_3_ catalyst. The obtained Pd/Al_2_O_3_ catalysts via the spray method were denoted as SP20, SP40 and SP60 according to their different support particle size. Palladium was also loaded onto the activated Al_2_O_3_ particle support (40~60 mesh) by the conventional incipient-wetness impregnation method followed by the same drying, calcination, and reduction process for comparison purpose, and the control sample was denoted as IM40.

### Characterization of Pd/Al_2_O_3_ catalysts

The X-ray diffraction (XRD) patterns of Pd/Al_2_O_3_ catalysts were obtained on a D/MAX-2004 X-ray powder diffractometer (Rigaku Corporation, Tokyo, Japan) with Ni-filtered Cu (*λ* = 0.15418 nm) radiation at 56 kV and 182 mA. Pd contents in these catalysts were determined with an inductively coupled plasma optical emission spectrometer (ICP-OES, 720-ES, Varian Inc, Palo Alto, CA, USA). X-ray photoelectron spectroscopy (XPS) measurements were conducted on an ESCALAB250 X-ray Photoelectron Spectrometer (Thermo Fisher Scientific Inc., Waltham, MA, USA) with an Al K anode (1486.6 eV, photon energy, 300 W). Their morphologies and microstructures were observed by a SUPRA35 Field Emission Scanning Electron Microscope (ZEISS, Germany) and a JEOL 2100 transmission electron microscope (JEOL Ltd., Tokyo, Japan) operating at 200 kV. Nitrogen adsorption-desorption measurements were performed with an Autosorb-1 Analyzer (Quantachrome Instruments, Boynton Beach, FL, USA). Their specific surface areas were calculated using the standard Brunauer-Emmett-Teller (BET) method and the mesopore size distributions were obtained by the Barrett-Joyner-Halenda (BJH) method from the desorption branch data of nitrogen adsorption isotherm. The degree of dispersion of Pd nanoparticles on these catalysts was determined by the CO chemisorption method with an Auto Chem II 2920 (Micromeritics instrument Ltd., Norcross, GA, USA). Briefly, 150 mg catalyst was activated in a 10% H_2_/Ar stream (50 mL · min^−1^) at 250 °C for 1 h. After purging with an He flow (50 mL · min^−1^) for 0.5 h, the sample was cooled down to 30 °C. Then, the CO chemisorption was conducted using the pulse titration model. The degree of dispersion and average size of Pd nanoparticles were calculated with the assumption that the CO/Pd stoichiometry at 1.

### Catalytic reduction experiments in the batch reactor

The catalytic reduction experiments in the batch reactor were carried out at room temperature (25 °C) and under the atmospheric pressure in a 250 mL three-necked flask reactor equipped with a mechanical Teflon stirrer. Briefly, 10 mg Pd/Al_2_O_3_ catalyst was grounded and added into the reactor containing 190 mL of DI water under mechanical stirring at 500 rpm followed by purging the reaction system with an H_2_ flow (200 mL·min^−1^) for 30 min. Then, 10 mL of bromate solution (8.0 mM) or methyl orange solution (1000 ppm) was added rapidly into the reactor and the suspension was continuously bubbled by an H_2_ flow (200 mL·min^−1^) under stirring. Samples were taken at selected time intervals and the catalyst was removed by centrifugation. The concentrations of bromate and bromide in the centrifugal supernatant were analyzed using Ion Chromatography (Dionex ICS 1100 Ion Chromatograph with a conductivity cell). A Dionex AS-22 column was used to separate bromate, bromide and other anions (4.2 mM carbonate and 1.4 mM bicarbonate buffer solutions as effluent). A 500 μL loop and a 25 μL loop were used to determine the concentrations of bromate and bromide at the microgram level and the milligram level, respectively. The detectable limitation for both bromate and bromide was lower than 1 ppb. The concentration of methyl orange was analyzed using UV-Vis spectrophotometer (UV-2550, SHIMADZU Corporation, Japan).

### Catalytic reduction experiments in the packed fixed-bed reactor

The continuous flow catalytic reduction experiments were carried out in a lab-prepared, packed fixed-bed tube reactor with the inner diameter of 10 mm and the bed height of 100 mm. The Pd/Al_2_O_3_ catalyst was packed into the tube and the experiment was conducted with upright flow mode at room temperature and under the atmospheric pressure. 800 mL · h^−1^ bromate solution and 1 mL · min^−1^ hydrogen were introduced into the packed bed reactor simultaneously. Both reverse osmosis (RO) water containing 0.078 mM (10 ppm) bromate and natural mineral water containing 0.4 μM (51 ppb) bromate were used for the experiment. For the reduction of methyl orange, RO water containing 50 ppm methyl orange were used, and the experiment conditions were the same as the treatment of bromate solution.

## Additional Information

**How to cite this article:** Gao, Y. *et al*. Creation of Pd/Al_2_O_3_ Catalyst by a Spray Process for Fixed Bed Reactors and Its Effective Removal of Aqueous Bromate. *Sci. Rep.* **7**, 41797; doi: 10.1038/srep41797 (2017).

**Publisher's note:** Springer Nature remains neutral with regard to jurisdictional claims in published maps and institutional affiliations.

## Supplementary Material

Supplementary Information

## Figures and Tables

**Table 1:** BET specific surface area, average pore size, and specific pore volume of Al_2_O_3_ supports and Pd/Al_2_O_3_ catalyst samples.

	Al_2_O_3_ Support (mesh)			Pd/Al_2_O_3_ Catalyst			
	20–40	40–60	60–100	SP20	SP40	SP60	IM40
BET Specific Surface Area (m^2^·g^−1^)	288.5	290.7	293.4	252.0	248.5	259.1	266.2
Average Pore Diameter (nm)	5.478	5.432	5.508	6.559	6.671	6.497	6.355
Specific Pore Volume (cm^3^·g^−1^)	0.3950	0.3949	0.4040	0.4132	0.4145	0.4208	0.4229

**Table 2:** The comparison of catalytic bromate reduction activity between SP60 sample and various Pd-based catalysts reported in literature.

Catalysts	Reaction Conditions	Initial Reaction Rate (mmol_Bromate_·h^−1^·g_Pd_^−1^)	Reference
0.88%Pd/Al_2_O_3_	0.4 mM Bromate, 0.05 g · L^−1^ Cat., 1 mL/mL_aq._ min H_2_, pH 6, RT	6001	This work
1.93%Pd/Al_2_O_3_	0.4 mM Bromate, 0.05 g · L^−1^ Cat., 0.2 mL/mL_aq._ min H_2_, pH 5.6, RT	554	[34]
2.2%Pd/MCN	0.78 mM Bromate, 0.03 g · L^−1^ Cat., 0.2 mL/mL_aq._ min H_2_, pH 5.6, RT	1818	[35]
1.5%Pd/ZSM5	0.078 mM Bromate, 0.5 g · L^−1^ Cat., 0.17 mL/mL_aq._ min H_2_, RT	25	[36]
0.104%Pd/Fe_3_O_4_	0.39 mM Bromate, 0.2 g · L^−1^ Cat., 0.5 mL/mL_aq._ min H_2_, pH 6, RT	5650	[40]
2.0%Pd/amino functionalized magnetic MCM-41	0.78 mM Bromate, 0.05 g · L^−1^ Cat., 0.2 mL/mL_aq._ min H_2_, pH 5.6, RT	1150	[41]
0.3%Pd/10%CNF/monolith	0.39 mM Bromate, 0.5 g · L^−1^ Cat., 0.42 mL/mL_aq._ min H_2_, RT	764	[44]
0.3%Pd/CNFs/Sintered Metal Fibers	0.39 mM Bromate, 1.67 g · L^−1^ Cat., 0.42 mL/mL_aq._ min H_2_, RT	470	[45]
0.3%Pd/CNFs/Carbon Cloth	0.39 mM Bromate, 0.83 g · L^−1^ Cat., 0.42 mL/mL_aq._ min H_2_, RT	861	[46]

**Table 3:** The kinetics parameters obtained in fitting the experimental data in Fig. 3.

	SP20	SP40	SP60	IM40
*K*_obs_ (min^−1^)	0.0717	0.0822	0.1255	0.0394
*R*^2^	0.999	0.994	0.980	0.999

**Table 4:** Water quality of test solutions used in the continuous flow catalytic bromate reduction experiments conducted in a lab-prepared, packed fixed-bed tube reactor.

	BrO_3_^−^	pH	Na^+^	Ca^2+^	Mg^2+^	K^+^	HCO_3_^−^	Cl^−^	NO_3_^−^	SO_4_^2−^	SiO_3_^2−^
RO Water	0.078 mM	~6.7	/	/	/	/	/	0.064 mM	0.084 mM	0.022 mM	/
Natural Mineral Water	0.40 μM	~7.7	3.479 mM	0.970 mM	0.445 mM	0.009 mM	2.797 mM	1.850 mM	0.084 mM	0.445 mM	0.341 mM
