# Supplementary material for: Creation of Pd/Al2O3 Catalyst by a Spray Process for Fixed Bed Reactors and Its Effective Removal of Aqueous Bromate
Source: Sci Rep. 2017 Feb 2;7:41797. doi: 10.1038/srep41797 (PMC5288787; doi:10.1038/srep41797)
Supplement: Supplementary Information [file srep41797-s1.doc]

**Creation of Pd/Al_2_O_3_ Catalyst by a Spray Process for Fixed Bed Reactors and Its Effective Removal of Aqueous Bromate**

Yu Gao^1,2,†^, Wuzhu Sun^1,3,†^, Weiyi Yang^1^, and Qi Li^1^,*

*^1^Environment Functional Materials Division*

*Shenyang National Laboratory for Materials Science*

*Institute of Metal Research, Chinese Academy of Sciences, Shenyang 110016, P. R. China*

*^2^Institute of Materials*

*China Academy of Engineering Physics, Mianyang 621900, P. R. China*

*^3^School of Materials Science and Engineering*

*Shandong University of Technology, Zibo 255000, P. R. China*

^†^These authors contributed equally to this work.

*Corresponding author.

E-mail address: [qili@imr.ac.cn](mailto:qili@imr.ac.cn) (Q. Li)

Phone: +86-24-83978028, Fax: +86-24-23971215.

Postal address: 72 Wenhua Road, Shenyang, Liaoning Province, 110016, P. R. China


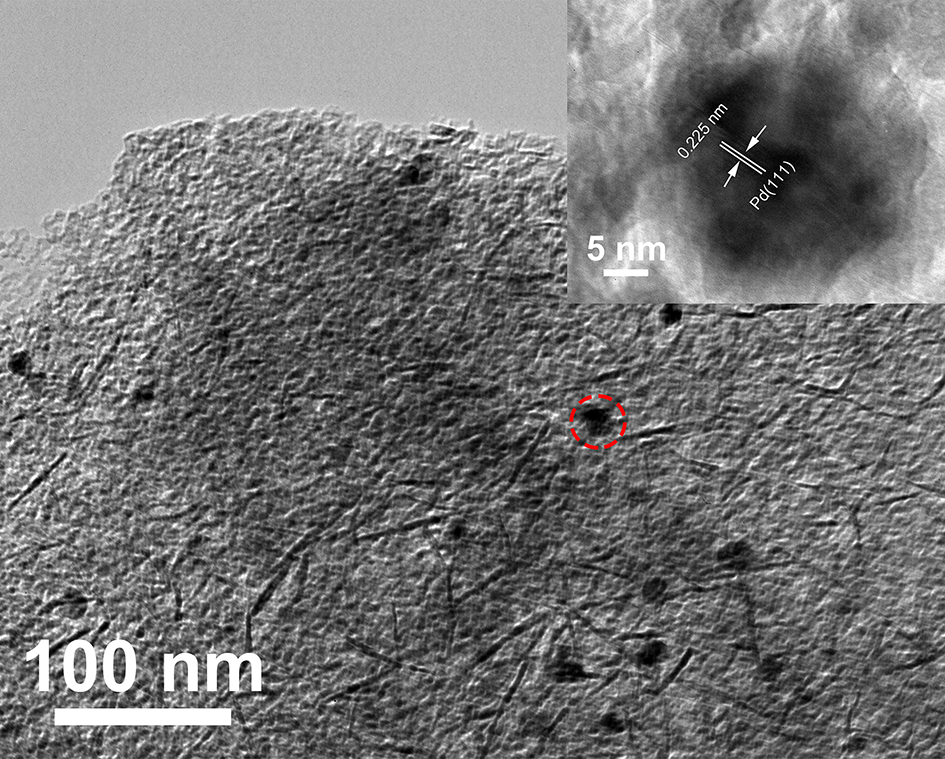


(a)


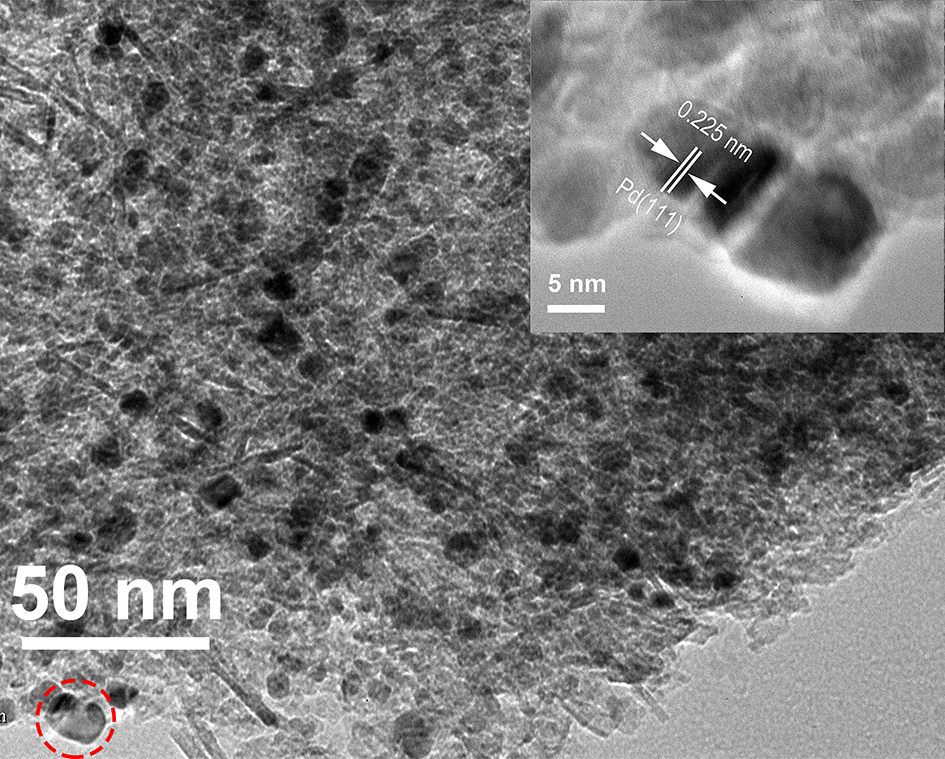


(b)


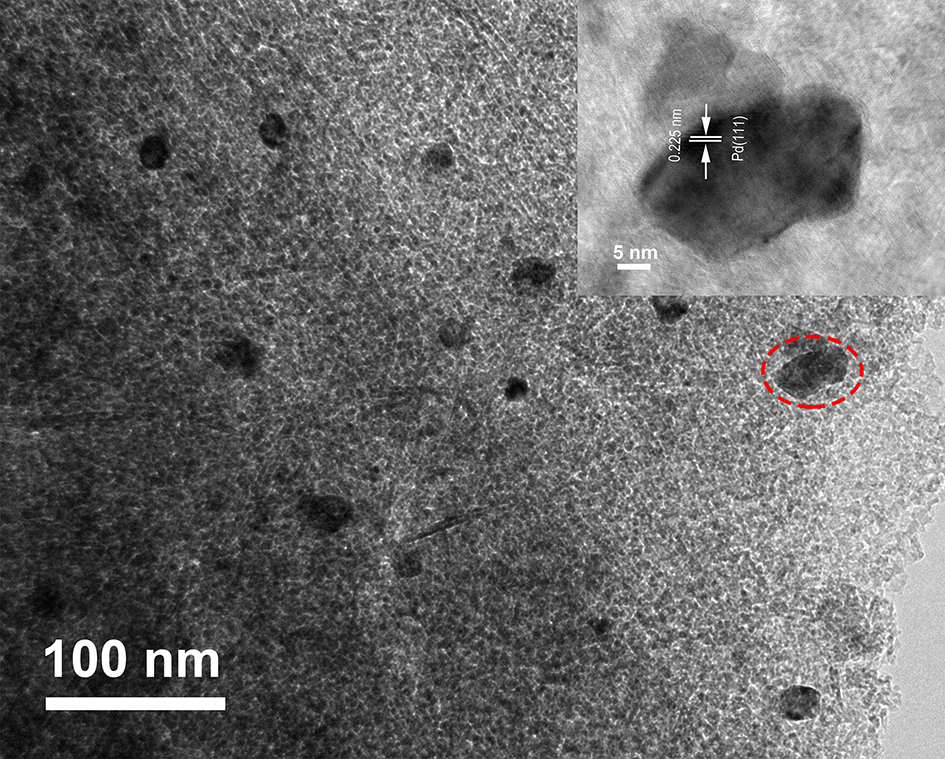


(c)

**Figure S1.** TEM images of (a) SP20, (b) SP40, and (c) IM40 samples (Note: Insert images in each figure show the corresponding HRTEM images of Pd nanoparticles with red circles).
